# Division in *Escherichia coli* is triggered by a size-sensing rather than a timing mechanism

**DOI:** 10.1186/1741-7007-12-17

**Published:** 2014-02-28

**Authors:** Lydia Robert, Marc Hoffmann, Nathalie Krell, Stéphane Aymerich, Jérôme Robert, Marie Doumic

**Affiliations:** 1 INRA, Micalis CNRS-UMR 1319, 78350 Jouy-en-Josas, France; 2 AgroParisTech, Micalis CNRS-UMR 1319, 78350 Jouy-en-Josas, France; 3 Paris Dauphine University, CNRS-UMR 7534, Place du maréchal De Lattre de Tassigny, 75775 Paris, France; 4 University of Rennes 1, CNRS-UMR 6625, Campus de Beaulieu, 35042 Rennes, France; 5 CNRS/UPMC University of Paris 6, FRE 3231, Laboratoire Jean Perrin LJP, 75005 Paris, France; 6 INRIA Paris-Rocquencourt, Domaine de Voluceau, BP 105, 781153 Le Chesnay, France; 7 UPMC University of Paris 6, JL Lions Lab., 4 place Jussieu, 75005 Paris, France

**Keywords:** Cell cycle, Bacteria, Division, Size control, Structured population equations, Numerical simulations, Nonparametric estimation

## Abstract

**Background:**

Many organisms coordinate cell growth and division through size control mechanisms: cells must reach a critical size to trigger a cell cycle event. Bacterial division is often assumed to be controlled in this way, but experimental evidence to support this assumption is still lacking. Theoretical arguments show that size control is required to maintain size homeostasis in the case of exponential growth of individual cells. Nevertheless, if the growth law deviates slightly from exponential for very small cells, homeostasis can be maintained with a simple ‘timer’ triggering division. Therefore, deciding whether division control in bacteria relies on a ‘timer’ or ‘sizer’ mechanism requires quantitative comparisons between models and data.

**Results:**

The timer and sizer hypotheses find a natural expression in models based on partial differential equations. Here we test these models with recent data on single-cell growth of *Escherichia coli*. We demonstrate that a size-independent timer mechanism for division control, though theoretically possible, is quantitatively incompatible with the data and extremely sensitive to slight variations in the growth law. In contrast, a sizer model is robust and fits the data well. In addition, we tested the effect of variability in individual growth rates and noise in septum positioning and found that size control is robust to this phenotypic noise.

**Conclusions:**

Confrontations between cell cycle models and data usually suffer from a lack of high-quality data and suitable statistical estimation techniques. Here we overcome these limitations by using high precision measurements of tens of thousands of single bacterial cells combined with recent statistical inference methods to estimate the division rate within the models. We therefore provide the first precise quantitative assessment of different cell cycle models.

## Background

Coordination between cell growth and division is often carried out by ‘size control’ mechanisms, where the cell size has to reach a certain threshold to trigger some event of the cell cycle, such as DNA replication or cell division [1]. As an example, the fission yeast *Schizosaccharomyces pombe* exhibits a size threshold at mitosis [2,3]. The budding yeast *Saccharomyces cerevisiae* also uses a size control mechanism that acts at the G1-S transition [4,5]. In contrast, in some cells such as those of early frog embryos, progression in the cell cycle is size independent and relies on a ‘timer’ mechanism [6].

Bacterial division is often assumed to be under size control but conclusive experimental evidence is still lacking and the wealth of accumulated data presents a complex picture. In 1968, building on the seminal work of Schaechter *et al*. and Helmstetter and Cooper, Donachie suggested that initiation of DNA replication is triggered when the bacterium reaches a critical size [7-9]. This provided the basis for a long-standing model of size control where cell size triggers replication initiation, which in turn determines the timing of division (see [10] and references therein). However, the coupling of replication initiation to cell mass has been repeatedly challenged [11-13]. In particular, on the basis of recent single-cell analysis, the team headed by N Kleckner proposed that replication initiation is more tightly linked to the time elapsed since birth than to cell mass [13,14]. In addition, the extent to which initiation timing affects division timing is unclear. In particular, variations in initiation timing are known to lead to compensatory changes in the duration of chromosome replication (see [15-17] and references therein). These studies argue against a size control model based on replication initiation. Another model postulates that size control acts directly on septum formation [18,19]. Nevertheless, the nature of the signals triggering the formation of the septal ring and its subsequent constriction are still unknown [17,20] and no molecular mechanism is known to sense cell size and transmit the information to the division machinery in bacteria.

Besides the work of Donachie, the assumption of size control in bacteria originates from a theoretical argument stating that such a control is necessary in exponentially growing cells to ensure cell size homeostasis, i.e. to maintain a constant size distribution through successive cycles. The growth of bacterial populations has long been mathematically described using partial differential equation (PDE) models. These models rely on hypotheses on division control: the division rate of a cell, i.e. the instantaneous probability of its dividing, can be assumed to depend either on cell age (i.e. the time elapsed since birth) or cell size. In the classical ‘sizer’ model, the division rate depends on size and not on age whereas in the ‘timer’ model it depends on age and not on size. Mathematical analysis of these models sheds light on the role of size control in cell size homeostasis. In particular, it has been suggested that for exponentially growing cells, a timer mechanism cannot ensure a stable size distribution [21,22]. Nevertheless, this unrealistic behavior of the timer mechanism is based on a biologically meaningless assumption, namely the exponential growth of cells of infinitely small or large size [23,24]. Cells of size zero or infinity do not exist and particularly small or large cells are likely to exhibit abnormal growth behavior. In conclusion, the mathematical arguments that were previously developed are insufficient to rule out a size-independent, timer model of bacterial division: quantitative comparisons between models and data are needed.

In the present study, we test whether age (i.e. the time elapsed since birth) or size is a determinant of cell division in *E. coli*. To do so, we analyzed two datasets derived from two major single-cell experimental studies on *E. coli* growth, performed by Stewart *et al.*[25] and Wang *et al.*[26]. Our analysis is based on division rate estimation by state-of-the-art nonparametric inference methods that we recently developed [27,28]. The two datasets correspond to different experimental setups and image analysis methods but lead to similar conclusions. We show that even though a model with a simple timer triggering division is sufficient to maintain cell size homeostasis, such a model is not compatible with the data. In addition, our analysis of the timer model shows that this model is very sensitive to hypotheses regarding the growth law of rare cells of very small or large size. This lack of robustness argues against a timer mechanism for division control in *E. coli* as well as in other exponentially growing organisms. In contrast, a model where cell size determines the probability of division is in good agreement with experimental data. Unlike the timer model, this sizer model is robust to slight modifications of the growth law of individual cells. In addition, our analysis reveals that the sizer model is very robust to phenotypic variability in individual growth rates or noise in septum positioning.

## Results and discussion

### Description of the data

#### Age and size distribution of the bacterial population

The results reported in this study were obtained from the analysis of two different datasets, obtained through microscopic time-lapse imaging of single *E. coli* cells growing in a rich medium, by Stewart *et al*. [25] and Wang *et al.*[26]. Stewart *et al*. followed single *E. coli* cells growing into microcolonies on LB-agarose pads at 30°C. The length of each cell in the microcolony was measured every 2 min. Wang *et al*. grew cells in LB: Luria Bertani medium at 37°C in a microfluidic setup [26] and the length of the cells was measured every minute. Due to the microfluidic device structure, at each division only one daughter cell could be followed (data *s*_*i*_: *sparse tree*), in contrast to the experiment of Stewart *et al.* where all the individuals of a genealogical tree were followed (data *f*_*i*_: *full tree*). It is worth noting that the different structures of the data *f*_*i*_ and *s*_*i*_ lead to different PDE models, and the statistical analysis was adapted to each situation (see below and Additional file 1). From each dataset (*f*_*i*_ and *s*_*i*_) we extracted the results of three experiments (experiments *f*_1_,*f*_2_ and *f*_3_ and *s*_1_,*s*_2_ and *s*_3_). Each experiment *f*_*i*_ corresponds to the growth of approximately six microcolonies of up to approximately 600 cells and each experiment *s*_*i*_ to the growth of bacteria in 100 microchannels for approximately 40 generations.

**Figure 1:**
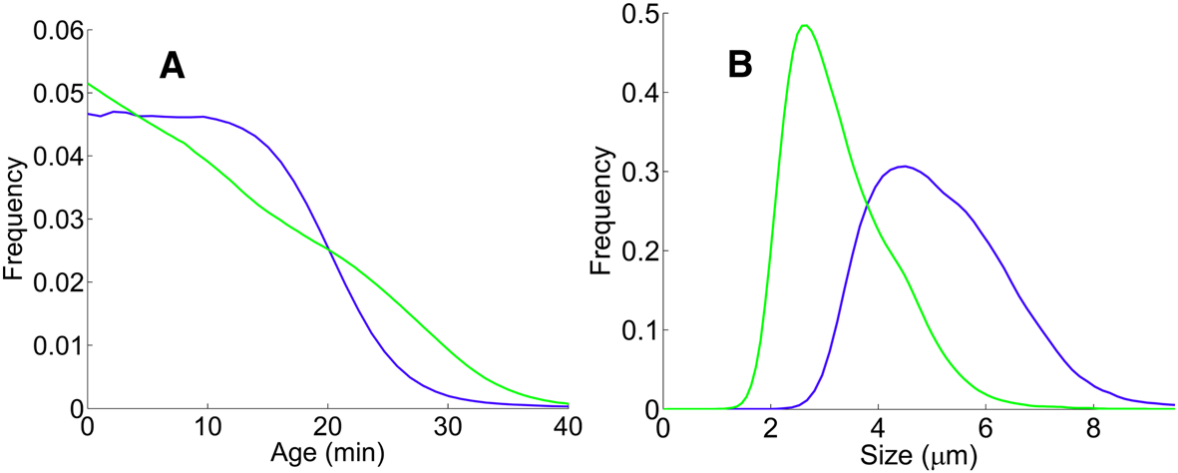
**Distributions of cell age and cell size.** Cell age **(A)** and cell size **(B)** distributions for a representative experiment of the *f*_*i*_ dataset from Stewart *et al.* (green) [25] and of the *s*_*i*_ dataset from Wang *et al.*[26] (blue).

Given the accuracy of image analysis, we do not take into account variations of cell width within the population, which are negligible compared to cell-cycle-induced length variations. Thus, in the present study we do not distinguish between length, volume and mass and use the term *cell size* as a catch-all descriptor. Cell age and cell size distributions of a representative experiment from each dataset are shown in Figure 1. These distributions are estimated from the age and size measurements of every cell at every time step of a given experiment *f*_*i*_ or *s*_*i*_, by using a simple kernel density estimation method (kernel estimation is closely related to histogram construction but gives smooth estimates of distributions, as shown in Figure 1, for instance; for details see the Methods and Additional file 1). As expected for the different data structures (full tree *f*_*i*_ or sparse tree *s*_*i*_) and different experimental conditions, the distributions for the two datasets are not identical. The age distribution is decreasing with a maximum for age zero and the size distribution is wide and positively skewed, in agreement with previous results using various bacterial models [29-31]. 

### Testing the timer versus sizer models of division

#### Age-structured (timer) and size-structured (sizer) models

The timer and sizer hypotheses are easily expressed in mathematical terms: two different PDE models are commonly used to describe bacterial growth, using a division rate (i.e. the instantaneous probability of division) depending either on cell age or cell size. In the age-structured model (Age Model) the division rate *B*_a_ is a function only of the age *a* of the cell. The density *n*(*t*,*a*) of cells of age *a* at time *t* is given as a solution to the Mckendrick–Von Foerster equation (see [32] and references therein):

(1) $$\frac{\partial }{\mathrm{\partial t}}n(t,a)+\frac{\partial }{\mathrm{\partial a}}n(t,a)=-B_{a}(a)n(t,a)$$

with the boundary condition

$$n(t,a=0)=2\int_{0}^{\infty }B_{a}(a)n(t,a)\text{da}$$

In this model, a cell of age *a* at time *t* has the probability *B*_a_(*a*)*dt* of dividing between time *t* and *t*+*dt*.

In the size-structured model (Size model), the division rate *B*_s_ is a function only of the size *x* of the cell. Assuming that the size of a single cell grows with a rate *v*(*x*), the density *n*(*t*,*x*) of cells of size *x* at time *t* is given as a solution to the size-structured cell division equation: [32]

(2) $$\begin{array}{c} \frac{\partial }{\mathrm{\partial t}}n(t,x)+\frac{\partial }{\mathrm{\partial x}}\left(v(x)n(t,x)\right)=-B_{s}(x)n(t,x)\\ \;+4B_{s}(2x)n(t,2x) \end{array}$$

In the Size Model, a cell of size *x* at time *t* has the probability *B*_s_(*x*)*dt* of dividing between time *t* and *t*+*dt*. This model is related to the so-called sloppy size control model [33] describing division in *S. pombe*.

For simplicity, we focused here on a population evolving along a full genealogical tree, accounting for *f*_*i*_ data. For data *s*_*i*_ observed along a single line of descendants, an appropriate modification is made to Equations (1) and (2) (see Additional file 1: Supplementary Text).

#### Testing the Age Model (timer) and the Size Model (sizer) with experimental data

In this study we tested the hypothesis of an age-dependent versus size-dependent division rate by comparing the ability of the Age Model and Size Model to describe experimental data. The PDE given by Equations (1) and (2) can be embedded into a two-dimensional age-and-size-structured equation (Age & Size Model), describing the temporal evolution of the density *n*(*t*,*a*,*x*) of cells of age *a* and size *x* at time *t*, with a division rate *B*_a,s_*a priori* depending on both age and size:

(3) $$\begin{array}{c} \left(\frac{\partial }{\mathrm{\partial t}}+\frac{\partial }{\mathrm{\partial a}}\right)n(t,a,x)+\frac{\partial }{\mathrm{\partial x}}\left(v(x)\;n(t,a,x)\right)=\\ \;-B_{\text{a,s}}(a,x)n(t,a,x) \end{array}$$

with the boundary condition

$$n(t,a=0,x)=4\int_{0}^{\infty }B_{\text{a,s}}(a,2x)n(t,a,2x)\text{da}$$

In this augmented setting, the Age Model governed by the PDE (1) and the Size Model governed by (2) are restrictions to the hypotheses of an age-dependent or size-dependent division rate, respectively (*B*_a,s_=*B*_a_ or *B*_a,s_=*B*_s_).

The density *n*(*t*,*a*,*x*) of cells having age *a* and size *x* at a large time *t* can be approximated as *n*(*t*,*a*,*x*)≈*e*^*λt*^*N*(*a*,*x*), where the coefficient *λ*>0 is called the *Malthus coefficient* and *N*(*a*,*x*) is the stable age-size distribution. This regime is rapidly reached and time can then be eliminated from Equations (1), (2) and (3), which are thus transformed into equations governing the stable distribution *N*(*a*,*x*). Importantly, in the timer model (i.e. *B*_a,s_=*B*_a_), the existence of this stable distribution requires that growth is sub-exponential around zero and infinity [23,24].

We estimate the division rate *B*_a_ of the Age Model using the age measurements of every cell at every time step. Likewise, we estimate the division rate *B*_s_ of the Size Model using the size measurements of every cell at every time step. Our estimation procedure is based on mathematical methods we recently developed. Importantly, our estimation procedure does not impose any particular restrictions on the form of the division rate function *B*, so that any biologically realistic function can be estimated (see Additional file 1: Section 4 and Figure S6). In Additional file 1: Figures S1 and S2, we show the size-dependent and age-dependent division rates *B*_s_(*x*) and *B*_a_(*a*) estimated from the experimental data. Once the division rate has been estimated, the stable age and size distribution *N*(*a*,*x*) can be reconstructed through simulation of the Age & Size Model (using the experimentally measured growth rate; for details see the Methods).

We measure the goodness-of-fit of a model (timer or sizer) by estimating the distance  between two distributions: the age-size distribution obtained through simulations of the model with the estimated division rate (as explained above), and the experimental age-size distribution. Therefore, a small distance  indicates a good fit of the model to the experimental data. To estimate this distance we use a classical metric, which measures the average of the squared difference between the two distributions. As an example, the distance between two bivariate Gaussian distributions with the same mean and a standard deviation difference of 10% is 17%, and a 25% difference in standard deviation leads to a 50% distance between the distributions. The experimental age-size distribution is estimated from the age and size measurements of every cell at every time step of a given experiment *f*_*i*_ or *s*_*i*_, thanks to a simple kernel density estimation method.

#### Analysis of single-cell growth

**Figure 2:**
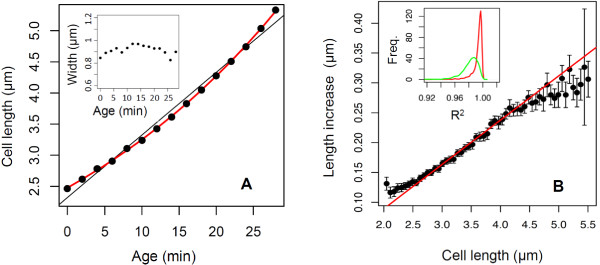
**Analysis of single-cell growth.(A)** Cell length vs cell age for a representative cell (black dots); exponential fit (red curve) and linear fit (black line). Inset: Cell width vs cell age for the same cell. **(B)** Increase in cell length during one time step (i.e. 1 min) as a function of cell length for *f*_*i*_ data. During the lifetime of a cell, the cell length is measured at each time step and the increase in cell length between successive time steps is calculated. Black dots are the average length increase for every cell of a given experiment *f*_1_, as a function of cell length; error bars are the average +/−2 SEM (standard error of the mean). The red line is a linear fit for lengths between 2.5 µm and 4.5 µm. Inset: For each single cell of *f*_1_, the evolution of cell length with age was fitted with a linear or an exponential function (as shown in panel A). We thus obtain a distribution of *R*^2^ coefficients corresponding to the linear (green) and exponential (red) fits.

As mentioned above, to avoid unrealistic asymptotic behavior of the Age Model and ensure the existence of a stable size distribution, assumptions have to be made on the growth of very small and large cells, which cannot be exactly exponential. To set realistic assumptions, we first studied the growth of individual cells. As expected, we found that during growth, a cell diameter is roughly constant (see inset in Figure 2A). Figure 2A shows cell length as a function of time for a representative cell, suggesting that growth is exponential rather than linear, in agreement with previous studies [25,26,34-36]. To test this hypothesis further, we performed linear and exponential fits of cell length for each single cell. We then calculated in each case the *R*^2^ coefficient of determination, which is classically used to measure how well a regression curve approximates the data (a perfect fit would give *R*^2^=1 and lower values indicate a poorer fit). The inset of Figure 2B shows the distribution of the *R*^2^ coefficient for all single cells for exponential (red) and linear (green) regressions, demonstrating that the exponential growth model fits the data very well and outperforms the linear growth model. We then investigated whether the growth of cells of particularly small or large size is exponential. If growth is exponential, the increase in length between each measurement should be proportional to the length. Therefore, we averaged the length increase of cells of similar size and tested whether the proportionality was respected for all sizes. As shown in Figure 2B, growth is exponential around the mean cell size but the behavior of very small or large cells may deviate from exponential growth. We therefore determined two size thresholds *x*_*min*_ and *x*_*max*_ below and over which the growth law may not be exponential (e.g. for the experiment *f*_1_ shown in Figure 2B, we defined *x*_*min*_=2.3 µm and *x*_*max*_=5.3 µm).

#### The age-size joint distribution of *E. coli* corresponds to a size-dependent division rate

**Figure 3:**
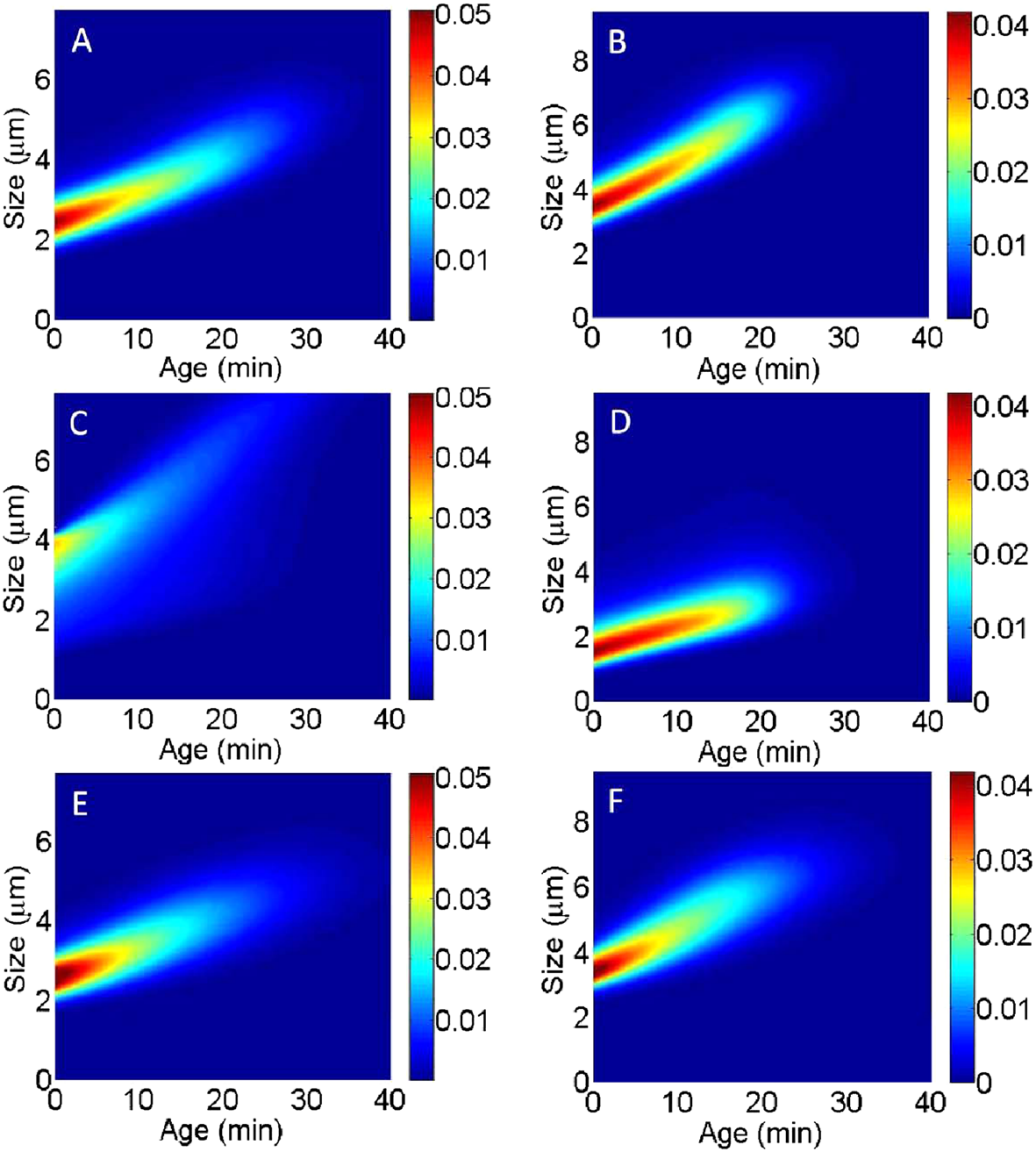
**Experimental and reconstructed age-size distributions for representative experiments from Stewart*et al.***[25]**(*f***_**1**_**) and Wang*et al.***[26]**(*s***_**1**_**).(A,B)** Experimental age-size distributions for representative experiments *f*_1_ (A) and *s*_1_ (B). The frequency of cells of age *a* and size *s* in the population is represented by the color at the point of coordinate *a* on the *x*-axis and *s* on the *y*-axis, according to the scale indicated to the right of the figure. **(C,D)** Reconstruction of the distributions using the Age Model (C: reconstruction of the data *f*_1_ shown in panel A; D: reconstruction of the data *s*_1_ shown in panel B). These reconstructed distributions were obtained from simulations with the Age Model using a division rate estimated from the data (C: from *f*_1_, D: from *s*_1_). The growth functions used for the simulations are detailed in the Methods section. **(E,F)** Reconstruction of the distributions using the Size Model (E: reconstruction of the data *f*_1_ shown in panel A; F: reconstruction of the data *s*_1_ shown in panel B). These distributions were obtained from simulations with the Size Model using a division rate estimated from the data (E: from *f*_1_, F: from *s*_1_) with an exponential growth function (see Methods).

We used both the Age Model and Size Model to fit the experimental age-size distributions, following the approach described above. The growth law below *x*_*min*_ and above *x*_*max*_ is unknown. Therefore, to test the Age Model, growth was assumed to be exponential between *x*_*min*_ and *x*_*max*_ and we tested several growth functions *v*(*x*) for *x*<*x*_*min*_ and *x*>*x*_*max*_, such as constant (i.e. linear growth) and polynomial functions. Figure 3 shows the best fit we could obtain. Comparing the experimental data *f*_1_ shown in Figure 3A (Figure 3B for *s*_1_ data) with the reconstructed distribution shown in Figure 3C (Figure 3D for *s*_1_ data) we can see that the Age Model fails to reconstruct the experimental age-size distribution and produces a distribution with a different shape. In particular, its localization along the *y*-axis is very different. For instance, for *f*_1_ data (panels A and C), the red area corresponding to the maximum of the experimental distribution is around 2.4 on the *y*-axis whereas the maximum of the fitted distribution is around 3.9. The *y*-axis corresponds to cell size. The size distribution produced by the Age Model is thus very different from the size distribution of the experimental data (experimental and fitted size distributions are shown in Additional file 1: Figure S9). 

As an additional analysis to strengthen our conclusion, we calculated the correlation between the age at division and the size at birth using the experimental data. If division is triggered by a timer mechanism, these two variables should not be correlated, whereas we found a significant correlation of −0.5 both for *s*_*i*_ and *f*_*i*_ data (*P*<10^−16^; see Additional file 1: Figure S7).

We used various growth functions for *x*<*x*_*min*_ and *x*>*x*_*max*_ but a satisfying fit could not be obtained with the Age Model. In addition, we found that the results of the Age Model are very sensitive to the assumptions made for the growth law of rare cells of very small and large size (see Additional file 1: Figure S3). This ultra-sensitivity to hypotheses regarding rare cells makes the timer model unrealistic generally for any exponentially growing organisms.

In contrast, the Size Model is in good agreement with the data (Figure 3: A compared to E and B compared to F) and allows a satisfactory reconstruction of the age-size structure of the population. The shape of the experimental and fitted distributions as well as their localization along the *y*-axis and *x*-axis are similar (size distributions and age distributions, i.e. projections onto the *y*-axis and *x*-axis, are shown in Additional file 1: Figure S8).

The quantitative measure of goodness-of-fit defined above is coherent with the curves’ visual aspects: for the Size Model the distance  between the model and the data ranges from 17% to 20% for *f*_*i*_ data (16% to 26% for *s*_*i*_ data) whereas for the Age Model it ranges from 51% to 93% for *f*_*i*_ data (45% to 125% for *s*_*i*_).

The experimental data has a limited precision. In particular, the division time is difficult to determine precisely by image analysis and the resolution is limited by the time step of image acquisition (for *s*_*i*_ and *f*_*i*_ data, the time step represents respectively 5% and 8% of the average division time). By performing stochastic simulations of the Size Model (detailed in Additional file 1: Section 6), we evaluated the effect of measurement noise on the goodness of fit of the Size Model. We found that noise of 10% in the determination of the division time leads to a distance  around 14%, which is of the order of the value obtained with our experimental data. We conclude that the Size Model fits the experimental data well. Moreover, we found that in contrast to the Age Model, the Size Model is robust with respect to the mathematical assumptions for the growth law for small and large sizes: the distance  changes by less than 5%.

### Size control is robust to phenotypic noise

Noise in the biochemical processes underlying growth and division, such as that created by stochastic gene expression, may perturb the control of size and affect the distribution of cell size. We therefore investigated the robustness of size control to such phenotypic noise. The Size Model describes the growth of a population of cells with variable age and size at division. Nevertheless, it does not take into account potential variability in individual growth rate or the difference in size at birth between two sister cells, i.e. the variability in septum positioning. To do so, we derived two PDE models, which are revised Size Models with either growth rate or septum positioning variability (see Additional file 1: Supplementary Text) and ran these models with different levels of variability.

#### Variability in individual growth rate has a negligible effect on the size distribution

For each single cell, a growth rate can be defined as the rate of the exponential increase of cell length with time [25,26]. By doing so, we obtained the distribution of the growth rate for the bacterial population (Additional file 1: Figure S4A). In our dataset this distribution is statistically compatible with a Gaussian distribution with a coefficient of variation of approximately 8% (standard deviation/mean =0.08).

**Figure 4:**
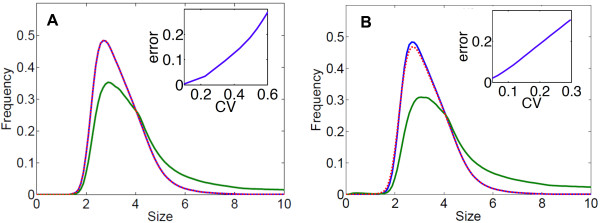
**Influence of the variability in individual growth rate and septum positioning on the cell size distribution.(A)** Size distributions simulated using the Size Model with the division rate *B*_s_ estimated from *f*_1_ data and an exponential growth (*v*(*x*)=*vx*). In blue: Simulations with the same growth rate *v*=0.0274 for every cell. Red dashed line: Simulations with individual growth rates distributed according to the experimentally observed distribution. Green line: Growth rates normally distributed with coefficient of variation CV =60*%*. Inset: Difference (i.e. normalized integrated squared error) between the size distribution simulated without variability and the distributions simulated with various levels of variability (normally distributed individual growth rates of CV between 10% and 60%). **(B)** Simulated size distributions using the Size Model with the same division rate *B*_s_ as in A and a constant growth rate *v*=0.0274. In blue: Simulations where division is perfectly symmetrical. Red dashed line: Simulations with a variable septum position distributed according to the experimentally observed distribution. Green line: Simulations with a normally distributed septum position with CV =30*%*. Inset: Difference between the size distribution simulated without variability in the septum position and the distributions simulated with various levels of variability (normally distributed septum position of CV between 5% and 30%). CV, coefficient of variation.

We recently extended the Size Model to describe the growth of a population with single-cell growth rate variability (the equation is given in Additional file 1: Section 5) [28]. We simulated this extended Size Model using the growth rate distribution of *f*_*i*_ data. The resulting size distribution is virtually identical to the one obtained without growth rate variability (Figure 4A, red and blue lines). Therefore, the naturally occurring variability in individual growth rate does not significantly perturb the size control. To investigate the effect of growth rate variability further, we simulated the model with various levels of noise, using truncated Gaussian growth rate distributions with coefficients of variation from 5 to 60%. We found that to obtain a 10% change in size distribution, a 30% coefficient of variation is necessary, which would represent an extremely high level of noise (Figure 4A, inset).

#### Variability in septum positioning has a negligible effect on size distribution

The cells divide into two daughter cells of almost identical length. Nevertheless, a slight asymmetry can arise as an effect of noise during septum positioning. We found a 4% variation in the position of the septum (Additional file 1: Figure S4B), which is in agreement with previous measurements [35,37-39]. To test the robustness of size control to noise in septum positioning, we extended the Size Model to allow for different sizes of the two sister cells at birth (the equation is given in Additional file 1: Section 5). We ran this model using the empirical variability in septum positioning (shown in Additional file 1: Figure S4B) and compared the resulting size distribution to the one obtained by simulations without variability. As shown in Figure 4B (comparing the red and blue lines), the effect of natural noise in septum positioning is negligible. We also ran the model with higher levels of noise in septum positioning and found that a three times higher (12%) coefficient of variation is necessary to obtain a 10% change in size distribution (Figure 4B inset, and Additional file 1: Figure S5).

## Conclusions

In the present study, we present statistical evidence to support the hypothesis that a size-dependent division rate can be used to reconstruct the experimental age-size distribution of *E. coli*. In contrast, this distribution cannot be generated by a timer model where the division rate depends solely on age. Even though the timer model can maintain cell size homeostasis, it is quantitatively incompatible with the observed size distribution. Our analysis of two different datasets shows the robustness of our conclusions to changes in experimental setup and image analysis methods. Our results therefore confirm the hypothesis of size control of division in *E. coli*. In addition, our analysis of the timer model shows that it is very sensitive to mathematical assumptions for the growth law of very rare cells of abnormal size, suggesting that this model is unrealistic for any exponentially growing organisms.

Noise in biochemical processes, in particular gene expression, can have a significant effect on the precision of biological circuits. In particular, it can generate a substantial variability in the cell cycle [5]. Therefore we investigated in bacteria the robustness of size control to noise in the single-cell growth rate and septum positioning, using appropriate extensions of the Size Model. We found that variability of the order of what we estimated from *E. coli* data does not significantly perturb the distribution of cell size. Therefore, in a natural population exhibiting phenotypic noise, the control of cell size is robust to fluctuations in septum positioning and individual growth rates. From a modeling perspective, this demonstrates that the simple Size Model is appropriate for describing a natural bacterial population showing phenotypic diversity.

Our approach is based on comparisons between PDE models and single-cell data for the cell cycle. Such comparisons were attempted a few decades ago using data from yeasts (e.g. [21,33]). Nevertheless, these interesting studies were hampered by the scarcity and poor quality of single-cell data as well as the lack of appropriate statistical procedures to estimate the division rate within the models. In contrast, we used high-precision measurements of tens of thousands cells in combination with modern statistical inference methods, which allowed us to assess quantitatively the adequacy of different models. We think this approach could prove successful in studying other aspects of the cell cycle, such as the coordination between replication and division or the molecular mechanisms underlying size control of division. Several different mechanisms involved in division control in bacteria have already been unraveled, in particular MinCD inhibition and nucleoid occlusion [40-42]. We believe that a better understanding of the relative roles played by MinCD inhibition and nucleoid occlusion in division control can be gained by analyzing the age-size distributions of *minCD* and nucleoid occlusion mutants. We are therefore currently performing time-lapse microscopy experiments to record the growth of such mutants.

## Methods

### Data analysis

The data of Stewart *et al.* contain the results of several experiments performed on different days, each of them recording the simultaneous growth of several microcolonies of the MG1655 *E. coli* strain on LB-agar pads at 30°C, with a generation time of approximately 26 min [25]. The first 150 min of growth were discarded to limit the effects of non-steady-state growth (cells undergo a slight plating stress when put on microscopy slides and it takes several generations to recover a stable growth rate). For the dataset obtained by Wang *et al.*, the MG1655 *E. coli* strain was grown in LB at 37°C in a microfluidic device with a doubling time of approximately 20 min. To avoid any effect of replicative aging such as described in [26], we only kept the first 50 generations of growth. In addition the first ten generations were discarded to ensure steady-state growth. Both datasets were generated by analyzing fluorescent images (the bacteria express the Yellow Fluorescent Protein) using two different software systems. For *s*_*i*_ data, cell segmentation was based on the localization of brightness minima along the channel direction (see [26]). In the same spirit, for *f*_*i*_ data, local minima of fluorescence intensity were used to outline the cells, following by an erosion and dilation step to separate adjacent cells (see [25]). To measure its length, a cell was approximated by a rectangle with the same second moments of pixel intensity and location distribution (for curved cells the measurement was done manually).

For both datasets we extracted data from three experiments done on different days. We did not pool the data together to avoid statistical biases arising from day-to-day differences in experimental conditions. Each analysis was performed in parallel on the data corresponding to each experiment.

#### Numerical simulations and estimation procedures

All the estimation procedures and simulations were performed using MATLAB. Experimental age-size distributions, such as those shown in Figure 3A,B, were estimated from the size and age measurements of every cell at every time step using the MATLAB kde2D function, which estimates the bivariate kernel density. This estimation was performed on a regular grid composed of 2^7^ equally spaced points on [ 0,*A*_*max*_] and 2^7^ equally spaced points on [ 0,*X*_*max*_], where *A*_*max*_ is the maximal cell age in the data and *X*_*max*_ the maximal cell size (for instance *A*_*max*_=60 min and *X*_*max*_=10 µm for the experiment *f*_1_, as shown in Figure 3A). To estimate the size-dependent division rate *B*_s_ for each experiment, the distribution of size at division was first estimated for the cell size grid [ 0,*X*_*max*_] using the ksdensity function. This estimated distribution was then used to estimate *B*_s_ for the size grid using Equation (20) (for *s*_*i*_ data) or (22) (for *f*_*i*_ data) of Additional file 1. The age-size distributions corresponding to the Size Model (Figure 3E,F) were produced by running the Age & Size Model (Equation (3) in the main text) using the estimated division rate *B*_s_ and an exponential growth function (*v*(*x*)=*vx*) with a rate *v* directly estimated from the data as the average of single-cell growth rates in the population (e.g. *v*=0.0274 min ^−1^ for the *f*_1_ experiment and *v*=0.0317 min ^−1^ for *s*_1_). For the Age & Size Model, we discretized the equation along the grid [ 0,*A*_*max*_] and [ 0,*X*_*max*_], using an upwind finite volume method described in detail in [43]. We used a time step:

$$\text{dt}=\frac{0.9}{\frac{2^{7}\times \max(v(x))}{X_{\text{max}}}+\frac{2^{7}}{A_{\text{max}}}}$$

meeting the CFL: Courant-Friedrichs-Lewy stability criterion. We simulated *n*(*t*,*a*,*x*) iteratively until the age-size distribution reached stability (|(*n*(*t*+*dt*,*a*,*x*)−*n*(*t*,*a*,*x*))|<10^−8^). To eliminate the Malthusian parameter, the solution *n*(*t*,*a*,*x*) was renormalized at each time step (for details see [43]).

The age-dependent division rate *B*_a_ for each experiment was estimated for the cell age grid [0,*A*_*max*_] using Equation (14) and (16) of Additional file 1. Using this estimated division rate, the age-size distributions corresponding to the Age Model (Figure 3C,D) were produced by running the Age & Size Model. As explained in the main text, we used various growth functions for small and large cells (i.e. for *x*<*x*_*min*_ and *x*>*x*_*max*_; between *x*_*min*_ and *x*_*max*_ growth is exponential with the same rate as for the Size Model). For instance for the fit of the experiment *f*_1_ shown in Figure 3C, for *x*<2.3 µm and *x*>5.3 µm, *v*(*x*)= max(*p*(*x*),0), with *p*(*x*)=−0.0033*x*^3^+0.036*x*^2^−0.094*x*+0.13. Likewise, for the fit of the experiment *s*_1_ shown in Figure 3D, for *x*<3.5 µm and *x*>7.2 µm, *v*(*x*)= max(*p*(*x*),0), with *p*(*x*)=−0.0036*x*^3^+0.063*x*^2^−0.33*x*+0.67. For each dataset the polynomial *p*(*x*) was chosen as an interpolation of the function giving the length increase as a function of length (shown in Figure 2B for *f*_1_ data).

Simulations of the extended size models with variability in growth rates or septum positioning (Equations (23) and (24) in Additional file 1) were performed as for the Age & Size Model, with an upwind finite volume scheme. To simulate Equation (23), we used a grid composed of 2^7^ equally spaced points on [ 0,*X*_*max*_] and 100 equally spaced points on [ 0.9*v*_*min*_,1.1*v*_*max*_], where *v*_*min*_ and *v*_*max*_ are the minimal and maximal individual growth rates in the data.

## Abbreviations

PDE: partial differential equation.

## Competing interests

The authors declare that they have no competing interests.

## Authors’ contributions

LR and MD conceived and designed the study, performed the analysis and drafted the manuscript. MH participated in the design of the study and helped to draft the manuscript. NK, SA and JR provided analytical tools. All authors read and approved the final manuscript.

## Supplementary Material

Additional file 1 Supplementary text and figures. Click here for file
