# Supplementary material for: Division in Escherichia coli is triggered by a size-sensing rather than a timing mechanism
Source: BMC Biol. 2014 Feb 28;12:17. doi: 10.1186/1741-7007-12-17 (PMC4016582; doi:10.1186/1741-7007-12-17)
Supplement: Additional file 1 — Supplementary text and figures. [file 1741-7007-12-17-S1.pdf]

Division in *Escherichia coli* is Triggered by a Size-sensing rather than a Timing Mechanism Supplementary Text

Lydia Robert Marc Hoffmann Nathalie Krell Stéphane Aymerich
Jérôme Robert Marie Doumic

After an overview of our statistical approach, we recall here how a probability density can be estimated from a sample of independent identically distributed random variables by kernel estimation methods [1]. We then explain in details for each model (the Age Model and the Size Model) and each data structure (f_i and s_i) how we estimate the division rate B. We then show that this estimation procedure allows reconstructing a division rate with sharp transitions. Finally, we describe the extended size models with variability in individual growth rate and septum positioning and we detail the stochastic simulations we performed in order to assess the impact of measurement noise on the goodness-of-fit of the models.

In our analysis, we distinguish two different data structures corresponding to the f_i and s_i datasets. For one microcolony of an experiment f_i, all the cells of the genealogical tree generated from a single ancestor are followed up to a given time. For each experiment f_i, we pooled the measurements done on every cell at every time point for all the microcolonies. In the case of s_i data, at each division only one daughter cell is followed, thus forming a single line of descendants along the genealogical tree of the population, which is followed up to a given time. Here again, for each experiment we pooled the data of all cells at all time points. In both cases, theoretical analysis shows that several lineages can be gathered to increase the total amount of observations [2]. The statistical analysis together with the PDE model have to be adapted to each data structure. This can be heuristically explained by the fact that when the cell population is followed along the whole genealogical tree untill a certain time (f_i data), a selection bias is introduced: cells that divide rapidly are overselected.

1 Statistical method

We estimate the age-size distribution $N(a, x)$ from the data using kernel density estimation methods (we call this the step $[K]$):

$$\text{data} \xrightarrow{[K]} N_{\text{empirical}}(\text{data}).$$

As detailed below, under the hypothesis of age-dependence or size-dependence, the division rates B_{a} and B_{s} can be statistically estimated from experimental measurements of respectively age and size of single cells in a population: we call this the step $[E]$. Once the division rate is estimated, the age-size distribution $N(a, x)$ can be reconstructed by simulation of the Age & Size Model (we call this the step $[R]$). All the estimation and reconstruction formulas depend on the data structure (i.e. f_i or s_i data).

We propose to measure the goodness-of-fit of model $\mathcal{M}$ = Age Model or Size Model by comparing two reconstruction schemes:

First, we estimate the division rate B from empirical data (step $[E]$) and reconstruct the function N by simulation of the Age & Size Model (step $[R]$):

$$\text{data} + \text{choice of } \mathcal{M} \xrightarrow{[E]} \text{estimator of } B \xrightarrow{[R]} N(\mathcal{M}, \text{data})$$

Alternatively, we directly reconstruct N from empirical data f_i or s_i through step $[K]$:

$$\text{data} \xrightarrow{[K]} N_{\text{empirical}}(\text{data}).$$

We can then define a goodness-of-fit measure for model $\mathcal{M}$ through the distance $\mathcal{D}(\mathcal{M}, \text{data})$

$$\mathcal{D}(\mathcal{M}, \text{data}) = \frac{\|N(\mathcal{M}, \text{data}) - N_{\text{empirical}}(\text{data})\|}{\|N_{\text{empirical}}(\text{data})\|} \tag{7}$$

where $\|\bullet\|$ denotes a norm (in our case, the squared-integrated norm over an appropriate domain for the age-size variable (a, x)). Thus $\mathcal{D}(\mathcal{M}, \text{data})$ measures the quality of reconstruction of the age-size distribution provided by model $\mathcal{M}$. We say that the Size Model dominates the Age Model as soon as $\mathcal{D}(\text{Size, data})$ is substantially smaller than $\mathcal{D}(\text{Age, data})$. In addition, we can visually compare $N(\mathcal{M}, \text{data})$ and $N_{\text{empirical}}(\text{data})$ for $\mathcal{M} = $ Age or Size.

2 Kernel density estimation (step $[K]$)

Kernel density estimation methods have for long proved to be accurate and they provide smooth functions approximating the underlying distribution, in contrast with histograms. For more details we refer to the textbook [1].

a. Estimating a density (step $[K]$ and tool for step $[E]$)

Let us assume that we have a sample $x_1, \cdots, x_n$ of n experimental measurements, each x_i being the realization of independent identically distributed random variables, whose underlying density is $N(x)$. This modelling assumption is justified in our case by the mathematical analysis and numerical simulations - see [2]: despite the dependence of cells taken on a genealogical tree, their distribution quickly converges toward a stationary regime characterized by an invariant probability measure. The sample can then be considered as if the cells were independent.

Kernel estimation methods define the following estimate $\hat{N}$ of N :

$$\hat{N}(x) := \frac{1}{n}\sum_{i=1}^{n} K_h * \delta_{x_i}(x) = \frac{1}{n}\sum_{i=1}^{n} K_h(x - x_i), \tag{8}$$

where δ_{x_i} denotes the Dirac mass in x_i and $K_h(x) := \frac{1}{h}K(\frac{x}{h})$ is a mollifier sequence, K being a function satisfying

$$K \in C_0^\infty(\mathbb{R}), \qquad \int K(x)dx = 1.$$

We used a Gaussian kernel K. In order to chose the optimal bandwidth h we used a data-driven method recently introduced by Goldenshluger and Lepski [3, 4, 5].

b. Estimating the derivative of a density (tool for step $[E]$)

It can be useful to estimate not only the density but also some functionals defined from this density: in our case, we need to estimate the derivative of N, denoted here $\frac{\partial}{\partial x}N(x)$ (see below; we keep the partial differential formalism to keep in mind the fact that N may depend on several variables). Keeping the notations of the previous paragraph, we may define an estimate $\widehat{\frac{\partial}{\partial x}N}$ by

$$\widehat{\frac{\partial}{\partial x}}N(x) := \frac{1}{n}\sum_{i=1}^{n} \frac{\partial}{\partial x}K_h * \delta_{x_i}(x) = \frac{1}{n}\sum_{i=1}^{n} \frac{\partial}{\partial x}(K_h)(x - x_i). \tag{9}$$

As for the previous section, we use the Goldenshluger and Lepski method to optimally select the bandwidth h. Note that this bandwidth will generally be larger than the optimal one selected for the estimation of the density, see [5].

3 Estimating the Division Rate (step $[E]$)

In the Age Model

In this section we derive formulas linking the age-dependent division rate B_{a} to the age distribution of the population $N(a)$. From these formulas, B_{a} can be estimated using the kernel density estimate of $N(a)$.

a. The single branch case (s_i data)

When a genealogical tree is fully observed up to a certain time as for f_i data, the PDE governing the Age Model is given by Equation [1] of the Main Text and its boundary condition, namely:

$$\frac{\partial}{\partial t}n(t,a) + \frac{\partial}{\partial a}n(t,a) = -B_{\mathrm{a}}(a)n(t,a), \tag{10}$$

$$n(t,a=0) = 2\int_0^\infty B_{\mathrm{a}}(a)n(t,a)da. \tag{11}$$

If a single line of ancestors is followed (the case of s_i data), Equation (10) remains unchanged, however the condition (11) for newborn cells must be changed into

$$n(t,a=0) = \int_0^\infty B(a)n(t,a)da, \tag{12}$$

since only one cell of age 0 is kept after each division. Simple calculations show that in this context, under a stationary regime, the age density of a cell picked along the branch has distribution $N(a)$ given by

$$N(a) = N(0)\exp\big(-\int_0^a B_{\mathrm{a}}(s)ds\big). \tag{13}$$

Solving (13) yields

$$B_{\mathrm{a}}(a) = -\frac{1}{N(a)}\frac{\partial}{\partial a}N(a). \tag{14}$$

As explained above, $N(a)$ and $\frac{\partial}{\partial a}N(a)$ can be estimated from empirical data by kernel methods. These estimates canthen be plugged into formula (14) to obtain an estimate of $B_{\mathrm{a}}(a)$.

b. The full tree case (f_i data)

Mathematical analysis shows that when the full genealogical tree is observed (f_i data), the approximation $e^{\lambda t}n(t,a) \approx N(a)$ is valid, with $\lambda > 0$ representing the *fitness* of the population. The stable distribution $N(a)$ has the explicit representation

$$N(a) = N(0)\exp\big(-\lambda a - \int_0^a B_{\mathrm{a}}(s)ds\big) \tag{15}$$

Thus, the formula (15) is for f_i data the analog of the formula (13) derived for s_i data. Formula (14) becomes in this case

$$B_{\mathrm{a}}(a) = -\frac{1}{N(a)}\Big(\frac{\partial}{\partial a}N(a) + \lambda N(a)\Big). \tag{16}$$

In the Size Model

In order to estimate the size-dependent division rate B_{s} we developed two different methods. The first one performs the estimation using the size distribution of the population, denoted N, whereas the second one uses the distribution of size at division, denoted g here. The kernel density method then provides estimates for these densities, which are plugged in analytical or implicit formulae (see below).

a. The single branch case (s_i data)

For the full tree case corresponding to f_i data, the Size Model is given by Equation (2) of the Main Text, namely:

$$\frac{\partial}{\partial t}n(t,x) + \frac{\partial}{\partial x}\big(v(x)n(t,x)\big) = -B_{\rm s}(x)n(t,x) + 4B_{\rm s}(2x)n(t,2x). \tag{17}$$

In the single branch case (for s_i data), since only one cell is kept after each division, the income term in the right-hand side of Equation (17) is divided by two, and this equation must be changed into

$$\frac{\partial}{\partial t}n(t,x) + \frac{\partial}{\partial x}\big(v(x)n(t,x)\big) = -B_{\rm s}(x)n(t,x) + 2B_{\rm s}(2x)n(t,2x). \tag{18}$$

The stable distribution $N(x)$ is solution to

$$\frac{\partial}{\partial x}\big(v(x)N(x)\big) = -B_{\rm s}(x)N(x) + 2B_{\rm s}(2x)N(2x). \tag{19}$$

Although no closed-form formula are available for the solution to (19) it is however possible to estimate $B_{\rm s}(x)$ from $N(x)$ via (19) by the methods developed in ([6, 7, 5]). Another method for estimating $B_{\rm s}$ uses the distribution of division size $g(x)$. The following explicit formula is established in [2] in the case of exponential growth ($v(x) = vx$):

$$B_{\rm s}(x) = \frac{vxg(x)}{\int_x^{2x} g(y)dy}, \tag{20}$$

from which consistent estimators can be derived. A similar but slightly more complex formula stands for a general growth rate $v(x)$.

b. The full tree case (f_i data)

In that case, the steady-state approximation $e^{\lambda t}n(t,x) \approx N(x)$ is valid, with $\lambda = v$ when $v(x) = vx$,. $N(x)$ is solution to

$$\lambda N(x) + \frac{\partial}{\partial x}\big(v(x)N(x)\big) = -B_{\rm s}(x)N(x) + 4B_{\rm s}(2x)N(2x). \tag{21}$$

As for the single branch case, although no closed-form formula is available for the solution to (21), it is possible to estimate $B_{\rm s}(x)$ from $N(x)$ via (21) by the methods developed in [5]. The following representation holds for the case $v(x) = vx$ (and a generalization is possible for general forms of $v(x)$)

$$B_{\rm s}(x) = \frac{vx^2g(x)}{\int_x^{2x} yg(y)dy}, \tag{22}$$

and the same subsequent methodology applies for estimating $B_{\rm s}(x)$.

4 Our procedure of division rate estimation allows to reconstruct sharp transitions

The division rate $B_{\rm s}(x)$ might present sharp transitions between several regimes, for instance due to specific responses of the cells of particularly small or large size. In order to demonstrate that our estimation procedure (described above) allows reconstructing the division rate in such a case, we performed a stochastic simulation of the Size Model (details of the stochastic simulations are presented in Section 6), using a division rate with sharp transitions. We then estimated the division rate from the simulated data using our estimation procedure (described above and in the main text). Figure S6 shows that the initial division rate (red dotted line) can be reconstructed with good precision (blue line), and the reconstructed function also presents sharp transitions.

5 Incorporating variability in the Size Model

a. Variability in individual growth rates

We recently introduced a stochastic model describing the growth of a population with single-cell growth rate variability by a piecewise deterministic Markov branching tree and demonstrated that the mean empirical measure follows a growth-fragmentation type PDE [2]. This mathematical analysis validates the use of this PDE as an extension of the Size Model describing the growth of cells with variability in individual growth rate. The equation is structured in both cell size and growth rate. It is based on exponential growth of single cells ($v(x) = vx$) with variable rate v and a size-dependent division rate:

$$\frac{\partial}{\partial t}n(t,x,v) + \frac{\partial}{\partial x}\big(vxn(t,x,v)\big) = -B(x)n(t,x,v) + 4\rho(v)\int_{v_{min}}^{v_{max}} B(2x)n(t,2x,v')dv', \tag{23}$$

where $n(t,x,v)$ is the density of cells of size x and growth rate v at time t. Like in the simple Size Model, $B(x)$ is the division rate: a cell has a probability $B(x)dt$ to divide during the time dt, giving birth to two cells of size $x/2$. The daughter growth rate v is distributed with a density ρ whose support is included in (v_{min}, v_{max}). When there is no variability, $\rho(v) = \delta_{v=\bar{v}}$ and integrating along v leads back to Equation (17) (with $\bar{v}$ replacing v in Equation (17)) for $n(t,x) = \int_{v_{min}}^{v_{max}} n(t,x,v)dv$.

b. Variability in septum positioning

In the same spirit, incorporating noise in septum positioning in the Size Model is described by the following growth-fragmentation equation:

$$\frac{\partial}{\partial t}n(t,x) + \frac{\partial}{\partial x}\big(v(x)n(t,x)\big) = -B(x)n(t,x) + 2\int_{x}^{\infty} B(y)n(t,y)k(y,x)dy. \tag{24}$$

This is a generalization of Equation (17): the only difference is that here, a dividing cell of size y can give birth to two cells of respective sizes $x \leq y$ and $y - x$ with a probability kernel $k(y, dx)$. Taking $k(y,dx) = \delta_{x=\frac{y}{x}}$ (*i.e.* perfectly symmetrical division) leads back to Equation (17).

6 Stochastic simulations

Here we first describe our general procedure for stochastic simulations of the Size Model. Then we detail the simulations we performed to assess the impact of noise in division time measurement and the simulations we performed to evaluate the estimation of a particular division rate with sharp transitions (described above in Section 4).

In a previous work [2], we performed calculations in a stochastic framework (with a size-dependent division rate) and recalled the classical following formula linking the probability $f(a, x_0)$ of dividing at age a given the size at birth x_0:

$$f(a,x_0) = B(x_0e^{va})e^{-\int_0^a B(x_0e^{vs})ds}. \tag{25}$$

Here cell growth is exponential with rate v, and B is the size-dependent division rate.

In our stochastic simulations, we start from a single cell with a given size at birth x_0. We then select at random its age at division a according to the density $f(\bullet, x_0)$ given by Formula (25) using a rejection sampling algorithm. From this age at division we calculate the size at division (x_oe^{va}) and the size of the two daughter cells at birth (assuming symmetrical division: $\frac{1}{2} \times x_oe^{va}$). This

process is then repeated for each cell of the next generation and the simulation is stopped after a certain number of generations. Variability in septum positioning and/or growth rate may easily be incoporated, see [2].

a. Fitting the Size Model to simulated data with noise in division time measurement

Measurement errors in the determination of the age and size at division may limit the goodness-of-fit of a model. In order to quantitatively assess this impact of measurement noise we performed stochastic simulations of the Size Model and added a white noise to the simulated data. Then, following the same approach as described in the main text for the experimental data, we estimated the division rate from this "noisy simulated data", simulated the Age & Size Model using this estimated division rate (as explained in the Methods in the main text) and assessed the distance $\mathcal{D}(\text{Size Model}, \text{data})$.

We performed 20 stochastic simulations with 9 generations, a division rate $B_{\rm s}(x) = x^7$, and exponential growth of the cells with rate $v = 1$. A 10 % white noise was added in the determination of the division time: the white noise ϵ was added to the simulated age at division and the corresponding size at division was multiplied by $e^{v\epsilon}$. As described in the main text, we found that with this noise, the obtained $\mathcal{D}(\text{Size Model}, \text{data})$ is around 14 % (average of the 20 simulations).

b. Simulations of the Size Model with a division rate exhibiting sharp transitions

We performed a stochastic simulation of 15 generations of the Size Model using the growth rate $v(x) = 0.0257x$ and the division rate $B(x)$ given by:

if $x < 3.9\mu m$, $B(x) = 4x^2/78^2$

if $x > 3.9\mu m$ and $x < 4.1\mu m$, $B(x) = 1.5x - 5.84$

if $x > 4.1\mu m$, $B(x) = 3x/41 + 0.01$

Then we used the simulated data to estimate the division rate according to the method described above and in the main text.

References

[1] Silverman, B.: Density Estimation for Statistics and Data Analysis. Monographs on Statistics and Applied Probability. Chapman and Hall, London, ??? (1986)

[2] Doumic, M., Hoffmann, M., Krell, N., Robert, L.: Statistical estimation of a growth-fragmentation model observed on a genealogical tree. ArXiv (2012)

[3] Goldenshluger, A., Lepski, O.: Uniform bounds for norms of sums of independent random functions. Ann. Probab. **39**, 2318–2384 (2011)

[4] Goldenshluger, A., Lepski, O.: Bandwidth selection in kernel density estimation: oracle inequalities and adaptive minimax optimality. Ann. Statist. **39**, 1608–1632 (2011)

[5] Doumic, M., Hoffmann, M., Reynaud, P., Rivoirard, V.: Nonparametric estimation of the division rate of a size-structured population. SIAM Num. Anal., (2012)

[6] Perthame, B., Zubelli, J.P.: On the inverse problem for a size-structured population model. Inverse Problems **23**(3), 1037–1052 (2007)

[7] Doumic, M., Perthame, B., Zubelli, J.P.: Numerical solution of an inverse problem in size-structured population dynamics. Inverse Problems **25**(electronic version), 045008 (2009)

Supplementary Figures

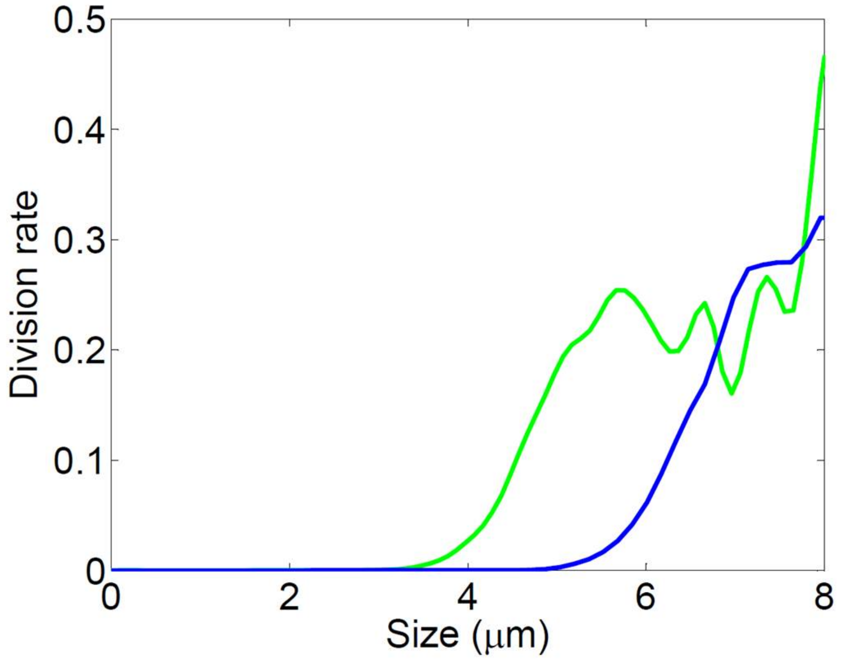

Figure S1: **Size-dependent division rate $B_{\mathbf{s}}$ estimated from f_1 data (green) and s_1 data (blue)**. Very long cells are rare and consequently the division rate estimation is imprecise for very large sizes (more than $5\mu m$ in f_i data or $7\mu m$ in s_i data.)

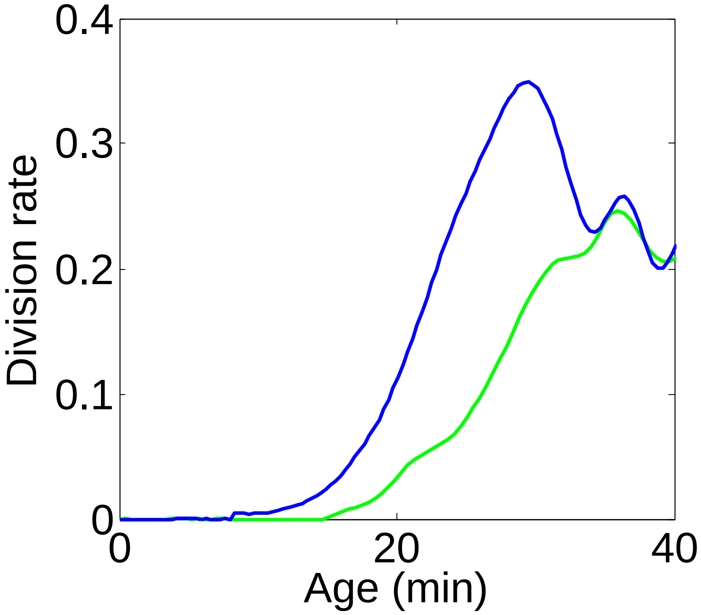

Figure S2: **Age-dependent division rate $B_{\mathbf{a}}$ estimated from f_1 data (green) and s_1 data (blue)**. Cells with large ages are rare and consequently the division rate estimation is imprecise for large ages.

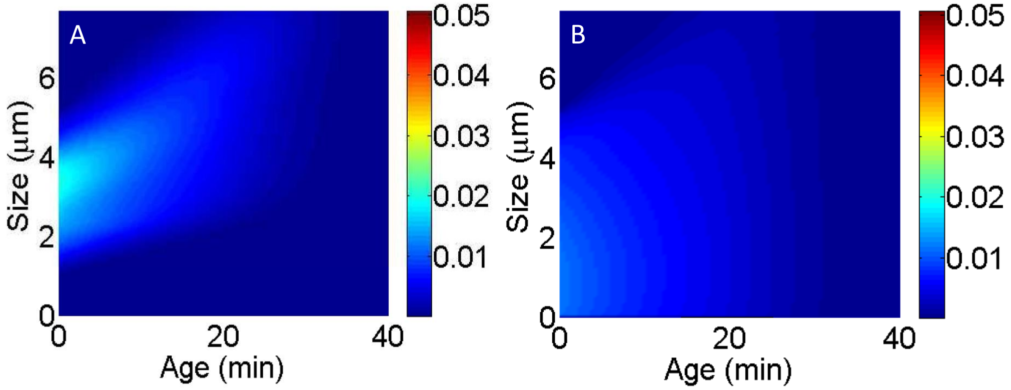

Figure S3: **Reconstructions of the Age-size distribution of the experiment f_1 with the Age Model using different growth functions** $v(x)$. A) Exponential growth for cell size between $2.3\mu m$ and $5.4\mu m$ ($v(x) = 0.0274x$) and linear growth elsewhere ($v(x) = 0.063$ for $x < 2.3$ and $v(x) = 0.148$ for $x > 5.4$). B) Exponential growth on a wider range : $v(x) = 0.0274x$ for $0.01 < x < 7\mu m$, $v(x) = 2.7 \times 10^{-4}$ for $x < 0.01\mu m$, and $v(x) = 0.19$ for $x > 7\mu m$. The color represents the frequency, according to the indicated scale. Comparing A and B and Figure 3C of the Main Text shows how much the Age Model is sensitive to the hypotheses on the growth law of the very rare cells of large and small size

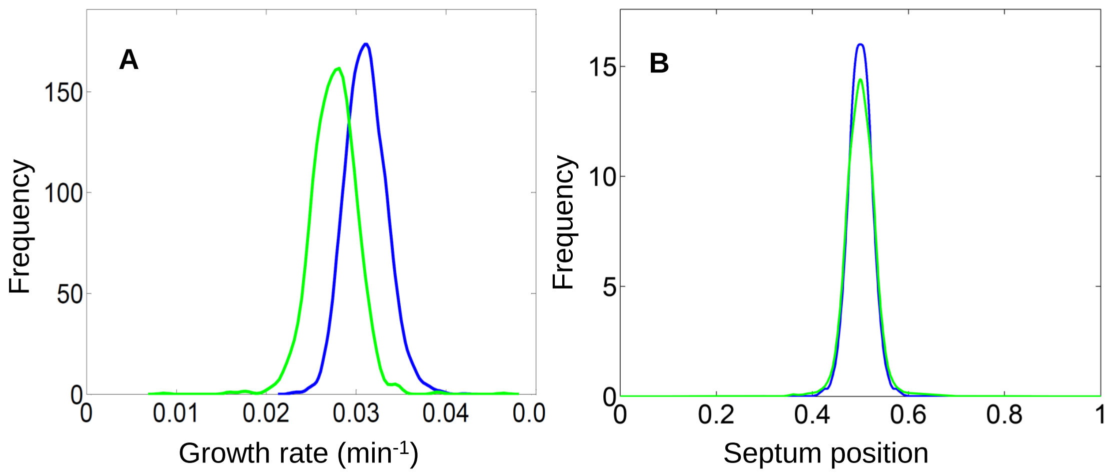

Figure S4: **Variability in growth rate and septum position among individual cells**.A) Growth rate distribution in a representative experiment from f_i data (green) and s_i data (blue). B) Distribution of septum position in a representative experiment from f_i data (green) and s_i data (blue). For each division event the septum position is computed as the length of one of the two daughter cells (randomly chosen) divided by the sum of the lengths of the two daughter cells.

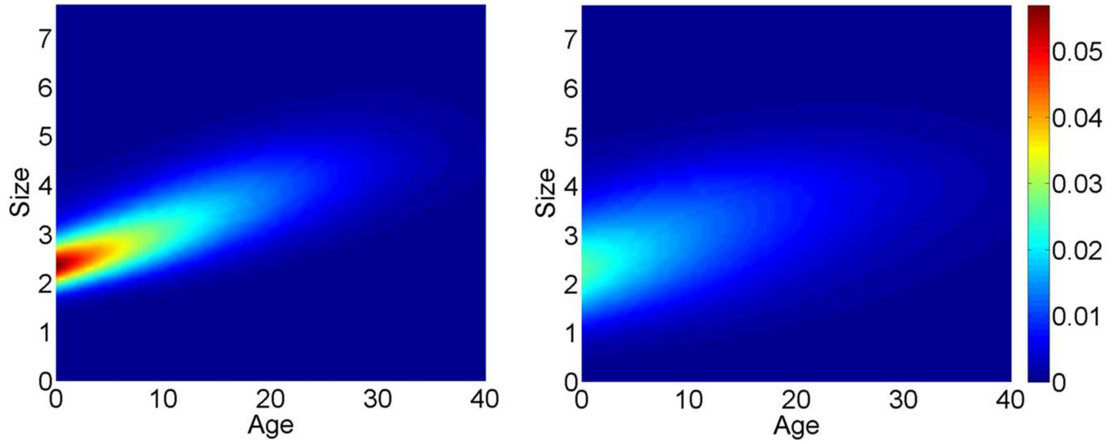

Figure S5: **Effect of variability in septum positioning on the age-size distribution**. A) Age-size distribution simulated from the extended Size Model with septum positioning variability, using the division rate estimated from f_1 data, exponential growth ($v(x) = 0.0274x$) and the septum position distribution measured from f_1 data. This result should be compared with Figure 3E, where no variability was supposed: the figures are virtually identical. B) Simulated age-size distribution with the same division rate and growth rate as in A) but with a large variability in the septum position, following a truncated Gaussian distribution with mean 0.5 and standard deviation CV=0.3.

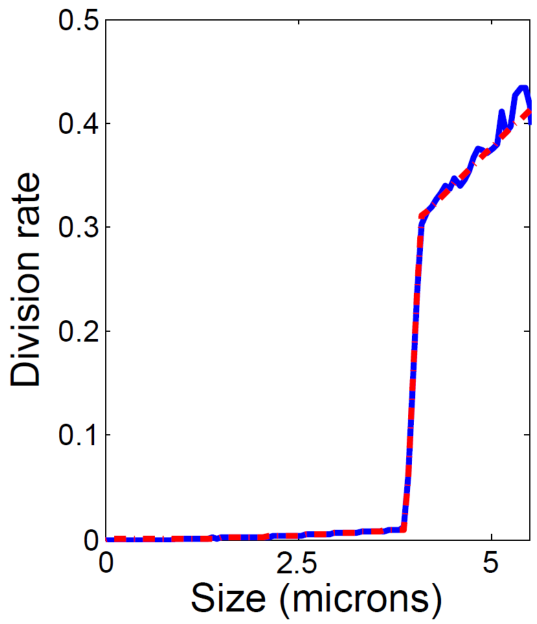

Figure S6: **Estimation of a size-dependent division rate with sharp transitions**. Red dotted line: size-dependent division rate used to perform stochastic simulations of the Size Model, as explained in the Supplementary Sections 4 and 6; blue line: division rate estimated from the simulated data.

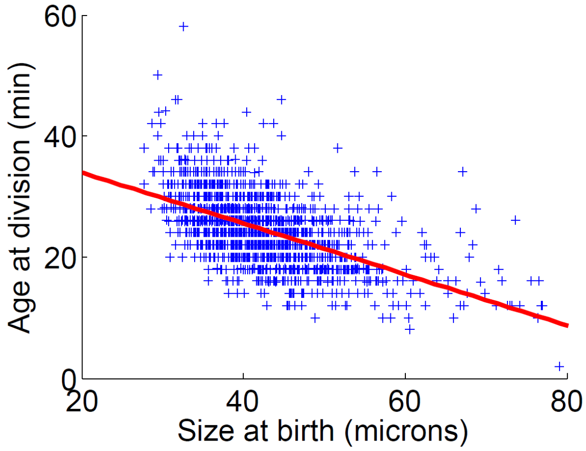

Figure S7: **Correlation between Size at birth and Age at division**. Each blue dot corresponds to a single cell of the experiment f_1; the red line is a linear regression

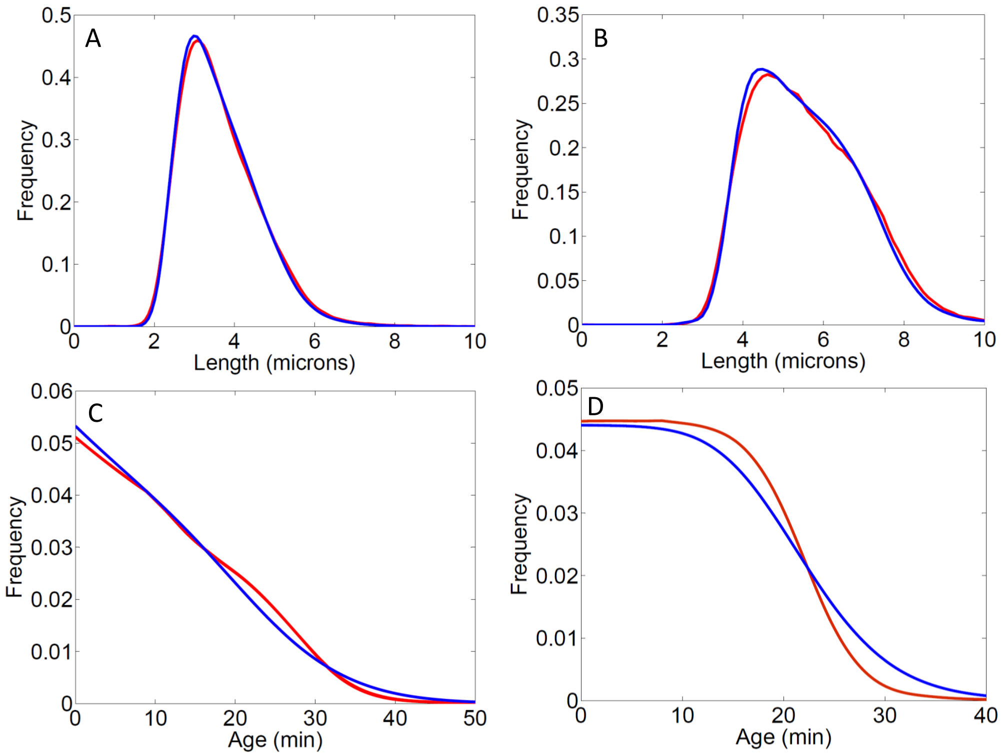

Figure S8: **Reconstruction of the experimental age distribution and size distribution with the Size Model for f_1 and s_1 data**. A) the experimental size distribution from f_1 experiment (red line) and its reconstruction using the Size Model (blue line); B) the experimental size distribution from s_1 experiment (red line) and its reconstruction using the Size Model (blue line); C) the experimental age distribution from f_1 experiment (red line) and its reconstruction using the Size Model (blue line); D) the experimental age distribution from s_1 experiment (red line) and its reconstruction using the Size Model (blue line);

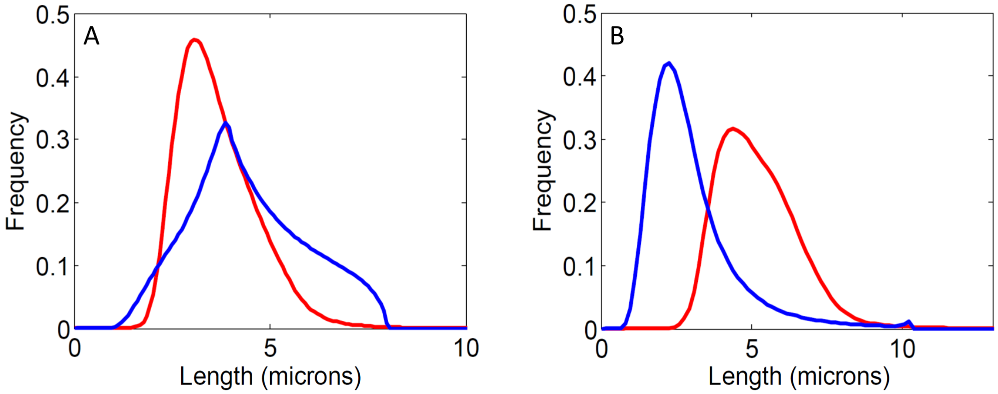

Figure S9: **Reconstruction of the experimental size distribution with the Age Model for f_1 and s_1 data**. A) the experimental size distribution from f_1 experiment (red line) and its reconstruction using the Age Model (blue line); B) the experimental size distribution from s_1 experiment (red line) and its reconstruction using the Age Model (blue line);
